# Specific Knockdown of OCT4 in Human Embryonic Stem Cells by Inducible Short Hairpin RNA Interference

**DOI:** 10.1002/stem.5

**Published:** 2009-04

**Authors:** Gaetano Zafarana, Stuart R Avery, Katie Avery, Harry D Moore, Peter W Andrews

**Affiliations:** Department of Biomedical Science, Centre for Stem Cell Biology, University of Sheffield, Western BankSheffield, United Kingdom

**Keywords:** Human embryonic stem cells, RNA interference, Inducible short hairpin RNAi, OCT4, β2-Microglobulin

## Abstract

Manipulation of gene function in embryonic stem cells by either over expression or downregulation is critical for understanding their subsequent cell fate. We have developed a tetracycline-inducible short hairpin RNA interference (shRNAi) for human embryonic stem cells (hESCs) and demonstrated doxycycline dose-dependent knockdown of the transcription factor OCT4 and the cell surface antigen β2-microglobulin. The induced knockdown of OCT4 resulted in rapid differentiation of hESCs with a significant increase in transcription of genes associated with trophoblast and endoderm lineages, the extent of which was controlled by the degree of induction. Transgene toxicity, which may occur in conditional over-expression strategies with hESCs, was not observed with wild-type Tet repressor protein. The system allows efficient, reversible, and long-term downregulation of target genes in hESCs and enables the generation of stable transfectants for the knockdown of genes essential for cell survival and self-renewal, not necessarily possible by nonconditional shRNAi methods.

## INTRODUCTION

Human embryonic stem cells (hESCs) are powerful tools for the in vitro study of early human development, and moreover provide a source of cells with therapeutic potential in regenerative medicine [1,2]. The molecular mechanisms controlling survival, self-renewal, and cell-fate decisions are not clearly defined, and strategies designed to control gene expression by either loss or gain-of-function are invaluable tools for their study. Problems associated with transfection efficiencies in hESCs [3,4] can hinder the use of transient systems to control gene expression, making the generation of stably-expressed transgenes a favorable option. However, difficulties may also arise if stably-expressed transgenes adversely regulate hESC survival, proliferation, or self-renewal. A solution is to place expression of the transgene under the constraints of an inducible mechanism. The tetracycline (Tet)-Off and Tet-On inducible expression systems [5] are most widely used to control transgene expression, though the Cre-ERT2 method is also an effective means of transgene regulation [6]. Inducible over expression of transgenes has been performed in hESCs using Tet [7,8] and Cre recombinase-based systems [9,10], but problems with the toxicity were encountered with constitutively expressed Tet activator and Cre in hESCs.

RNA interference (RNAi) is now established as a powerful tool for specific gene knockdown in mammalian cells [11]. One important advance has been the development of short hairpin RNA interference (shRNAi) systems driven by RNA polymerase III promoters [12–15]. These vector-based approaches permit long-term knockdown cell lines to be established, although it is only possible to use them transiently in hESCs for the knockdown of self-renewal associated genes such as OCT4 and SPA1 [4,16,17]. Conditional knockdown of target genes in other mammalian cells has been achieved using a doxycycline-inducible version of the H1 promoter in the pSUPER vector [15], termed pSUPERIOR, [18]. In this system, cells are required to express wild-type Tet repressor (TetR) protein and not the tetracycline-controlled transactivator protein (*tTA*) or the reverse tetracycline-controlled transactivator (*rtTA*). The *tTA* and *rtTA* proteins that are utilized by the Tet-Off and Tet-On systems, respectively, are based on fusions of the TetR protein to the transactivating domain of VP16 [19,20]. This transactivating domain is believed to be responsible for their toxicity in sensitive hESCs.

We have now used the TetR pSUPERIOR system to generate conditional shRNAi knockdowns of target genes in hESCs. By generating a stable hESC line constitutively expressing TetR under the control of the pCAG promoter [21,22], we generated clones that induced specific and dose-dependent knockdown of β2-microglobulin (β2M) and OCT4. The latter was targeted as an example of a gene required for the self-renewal of hESCs [23]. Downregulation of OCT4 results in differentiation of murine ESCs toward trophectoderm [24,25], a result that correlates with the differentiation of hESCs subjected to OCT4 RNAi toward trophoblast and endoderm lineage [4,16,26,27]. Using our inducible shRNAi system, we also observed dramatic differentiation of cells following induction of OCT4 knockdown, with an increase in expression of trophoblast and mesoderm-associated gene transcripts.

## MATERIALS AND METHODS

### Plasmid Construction

#### TetR Nuclear Localization Signal-pCAG

Wild-type TetR cDNA, containing an N-terminus SV40 nuclear localization signal (nls) (a kind gift of F.G. Grosveld), was excised from the pBSKS plasmid by XhoI and NotI digestion and inserted into the XhoI and NotI sites of pCAGeGFP [22] in which XhoI/NotI digestion excises the green fluorescent protein fragment.

#### pSUPERIOR-Target Hairpin

Target sequences were designed with an online small interfering RNA (siRNA) design tool (Ambion, Austin, TX, http://www.ambion.com). To construct the hairpin vectors, oligonucleotides (MWG Biotech, Ebersberg, Germany, http://www.mwg-biotech.com) were annealed by combining equal volumes of the sense and antisense hairpin oligonucleotides (100 μM in water). The mix was incubated in a water bath at 95°C for 5 minutes and allowed to cool to room temperature. The annealed oligonucleotides were designed to carry BglII and XhoI compatible overhangs enabling them to be cloned into the BamHI, XhoI linearized vector pSUPERIOR.neo (Oligoengine). Oligonucleotides were as follows:

- OCT4: 5′-GATCCGGATGTGGTCCGAGTGTGGTTCAAGAGACCACACTCGGACCACATCCTTTTTTC-3′ and 5′-TCGAGAAAAAAGGATGTGGTCCGAGTGTGGTCTCTTGAACCACACTCGGACCACATCCG-3′;
- β2M: 5′-GATCCGGACTGGTCTTTCTATCTCTTCAAGAGAGAGATAGAAAGACCAGTCCTTTTTTC-3′ and 5′-TCGAGAAAAAAGGACTGGTCTTTCTATCTCTCTCTTGAAGAGATAGAAAGACCAGTCCG-3′.

To confirm that cloned fragments were of the correct sequence, we used the following sequencing primer: 5′-AGAATTCGAACGCTGACGTC-3′.

### Cell Culture and Generation of Stable Transfectants

The Shef4 cell line was used throughout and exhibits standard morphological and surface marker characteristics of hESCs and a normal 46XY karyotype [28]. This cell line is one of several generated under license and guidelines of the Human Fertilization and Embryology Authority at the Centre for Stem cell Biology (University of Sheffield, U.K.). Shef4 has been independently validated and accepted by the U.K. Stem Cell Bank for distribution to the science community. hESCs were maintained in hESC medium comprising knockout-Dulbecco's modified Eagle's medium (DMEM) supplemented with 20% knockout serum replacement, 1% nonessential amino acids, 1 mM l-glutamine, 4 ng/ml basic fibroblast growth factor (all from Invitrogen, Carlsbad, CA, http://www.invitrogen.com), and 0.1 mM β-mercapto-ethanol (Sigma-Aldrich, St. Louis, MO, http://www.sigmaaldrich.com) at 37°C under a humidified atmosphere of 5% CO_2_ in air. Cells were passaged weekly by manual dissection: Following digestion for 5 minutes with 1 mg/ml collagenase type IV (Invitrogen), colonies were excised using the end of a 1 ml extended fine tip disposable pipette (Alpha Laboratories, Hampshire, United Kingdom, http://www.alphalabs.co.uk). Cells were seeded on feeder layers of mitotically inactivated mouse embryonic fibroblasts (MEFs) (∼7.2 × 10^3^ cells per cm^2^). MEFs were grown in DMEM (Invitrogen) supplemented with 10% heat inactivated fetal calf serum (FCS; Invitrogen) and mitotically inactivated by incubation with 10 μg/ml Mitomycin C (Sigma-Aldrich) for 3 hours. The FCS batch was prequalified to be free of doxycycline and tetracycline to prevent any nonexperimentally-induced transgene expression.

To generate stable transfected cell lines, collagenase digested hESC colonies were broken into small clumps and recovered from feeders by disruption in situ with 3 mm glass beads. Approximately 1 million cells were incubated with 10 μg vector (both TetRnls-pCAG and pSUPERIOR vectors were linearized by ScaI) and electroporated using the EasyjecT Optima electroporator (EquiBio) at 240 V, 1,050 μF, and resistance set to infinity. Transfectants were plated on mitotically inactivated MEF21 neomycin/puromycin-resistant fibroblasts in 10-cm^2^ Nunclon Δ surface Petri dishes (Nunc, Rochester, NY, http://www.nuncbrand.com). MEF21 is a spontaneously immortalized mouse fibroblast cell line derived from primary mouse fibroblasts derived in house and subsequently stably transfected to express G418 and puromycin resistance markers (data not published). Colonies of transfected cells were allowed to establish for 4-6 days of culture before antibiotic selection. TetRnls-pCAG transfectants were selected with hESC medium containing puromycin (Sigma-Aldrich) at 1 μg/ml and pSUPERIOR transfectants with G418 (Invitrogen) at 10 mg/ml. Once stable transgene expressing clones were generated, they were cultured on regular MEFs in hESC medium without antibiotics. Karyotype analysis was performed routinely to confirm euploidy of transfectants, using standard G-banding techniques as described previously [29].

### Northern Blotting Analysis

Northern analysis was performed to confirm hairpin transcription [30]. The sequences of the probes were as follows:

- Transfer RNA valine (loading control): 5′-GAACGTGATAACCACTACACTACGGAAACCCTATAGTGAGTCGTATTAGGCGGGAACCGCCTAATACGACTCACTATAGG-3′;
- OCT4: 5′-GGATGTGGTCCGAGTGTGG-3′.

### Immunoblotting

Cells were lysed by sonication in ice cold phosphate buffered saline containing 1% Nonidet P-40, 0.5% sodium deoxycholate, 0.1% SDS, 1 mM dl-dithiothreitol, 1 mM EDTA, 4 mM Na_3_V0_4_, 4 mM NaF, 1 mM phenylmethylsulfonyl fluoride, 1× final concentration Complete Protease inhibitor (Roche Diagnostics, Basel, Switzerland, http://www.roche-applied-science.com). Fifteen micrograms of extracted protein was fractionated by sodium dodecyl sulfate polyacrylamide gel electrophoresis on a 12.5% acrylamide gel and electroblotted onto a polyvinylidene difluoride membrane (Amersham Biosciences, Piscataway, NJ, http://www.amersham.com). Membranes were stained with either of the following antibodies: ab6276, anti-β-actin (Abcam, Cambridge, U.K., http://www.abcam.com), sc-9081, anti-Oct3/4 (Santa Cruz Biotechnology Inc., Santa Cruz, CA, http://www.scbt.com) or TET-02, anti-TetR (MoBiTec, Göttingen, Germany, http://www.mobitec.de), with sc-2005, goat anti-mouse and sc-2955, chicken anti-rabbit horseradish peroxidase (HRP)-conjugated secondary antibodies (Santa Cruz). HRP was detected using Supersignal West Pico chemiluminescent substrate (Pierce, Rockford, IL, http://www.piercenet.com) and visualized with Hyperfilm ECL film (Amersham Biosciences).

### Immunocytochemistry

Cells were fixed with 4% paraformaldehyde for and permeabilized using buffer containing phosphate buffered saline (PBS), 10% FCS, and 0.1% Triton X-100. Staining was performed using sc-5279, mouse monoclonal anti-Oct3/4 (Santa Cruz) and antibody localization was determined by M30801, Goat anti-mouse IgG + IgM (H + L) fluorescein isothiocyanate (FITC)-conjugated secondary antibody (Caltag Laboratories, Carlsbad, CA, http://www.invitrogen.com). Fluorescence was detected using an Olympus CKX41 microscope, and images were captured using a Nikon DS-L1 camera.

### Flow Cytometry

Single cells were harvested by trypsinization and resuspended in wash buffer (PBS with 5% FCS), and 100 μl aliquots containing 1 × 10^5^ cells were incubated with primary antibody for 1 hour. P3X63Ag8 (negative control) [31], anti-stage-specific embryonic antigen-3 (SSEA3) [32], and TRA-1-60 [33] antibodies were derived in-house from hybridoma supernatants as described earlier [34]. After washing by centrifugation, cells were incubated with goat anti-mouse IgG + IgM (H + L) FITC-conjugate (Caltag Laboratories) at 1:200 for 1 hour and analyzed after washing by flow cytometry using an ADP CyAn cytometer (Beckman Coulter, Fullerton, CA, http://www.beckmancoulter.com).

### Reverse Transcription-Polymerase Chain Reaction

For standard reverse transcription-polymerase chain reaction (RT-PCR) procedures, total RNA was extracted using TRIzol Reagent (Invitrogen) and DNAseI treated using the TURBO DNA-*free* kit (Ambion). For real time RT-PCR procedures, total RNA was extracted using the RNeasy Plus Mini Kit (Qiagen, Hilden, Germany, http://www1.qiagen.com) and QIA shredder (Qiagen). cDNA was synthesized using RevertAid H Minus M-MuLV Reverse Transcriptase (Fermentas, International Inc., Ontario, Canada, http://www.fermentas.com) and primers oligo-dT [16] and oligo-dN [6]. *Taq* DNA polymerase (Invitrogen) was used to amplify cDNA products, which were fractionated on 2% Tris/Borate/EDTA agarose gel and visualized under a UV source following ethidium bromide (Sigma-Aldrich) staining. Band intensities were analyzed using GeneTools software v3.07 (SynGene, Cambridge, United Kingdom, http://www.syngene.com). Amplification for real time RT-PCR was performed using SYBR Green JumpStart Taq ReadyMix (Sigma-Aldrich) and an iCycler (Bio-Rad, Hercules, CA, http://www.bio-rad.com) for signal detection. Expression of specific genes was compared with that of hypoxanthine-guanine phosphoribosyl transferase using the Genex algorithm (Bio-Rad) based on the ΔΔCt method [35]. An annealing temperature of 58°C was used in all reactions, using primers listed in Table 1.

**Table 1:** Primers used in polymerase chain reaction

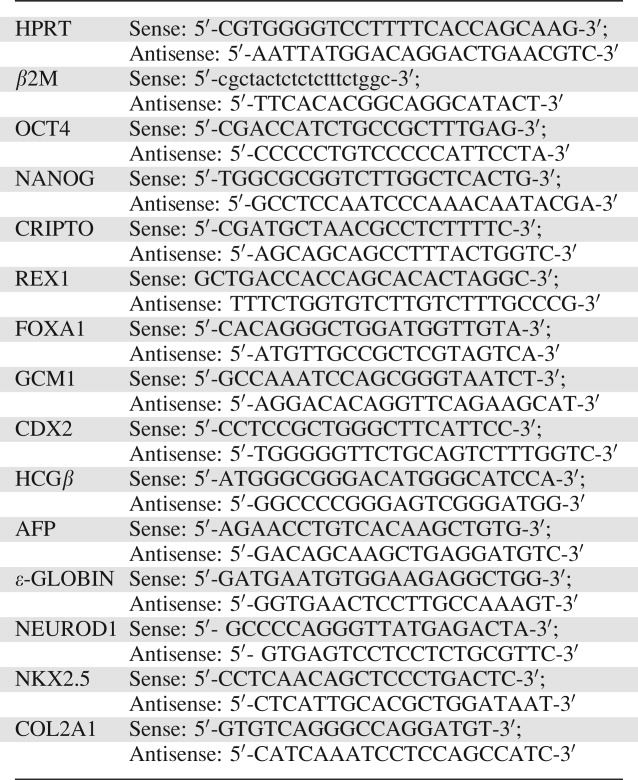

Abbreviations: AFP, alpha-fetoprotein; β2M, β2-microglobulin; HPRT, hypoxanthine-guanine phosphoribosyl transferase.

## RESULTS

### The Shef4 TetR5 hESC Line Constitutively Expressed TetR Protein

**Figure 1:**
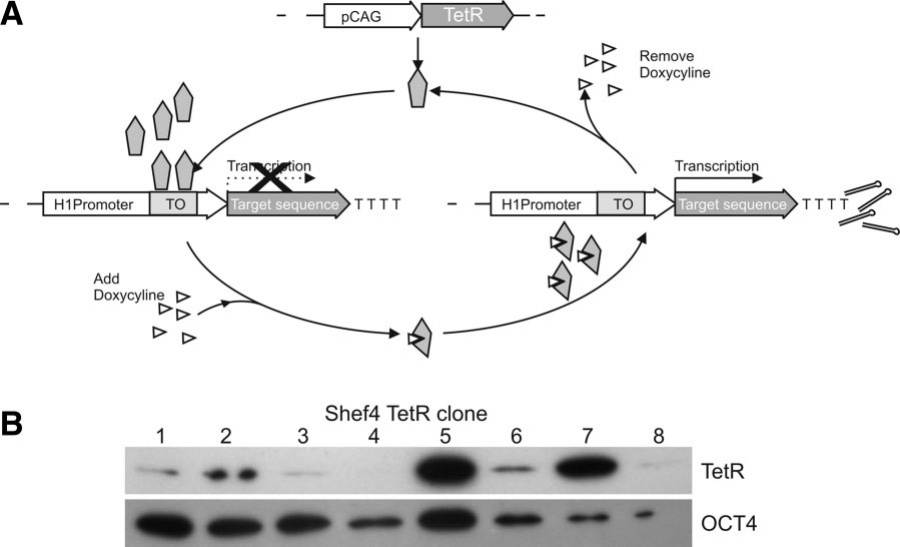
TetR-based inducible short hairpin RNA interference system. **(A):** Graphical overview of the system (based on tetracycline [Tet] systems manual; http://www.clonetech.com). Constitutively expressed TetR protein binds to the TO sequence within the H1 promoter, blocking expression of the target sequence hairpin. On addition, doxycycline binds to TetR protein, resulting in a conformational change that renders the TetR unable to bind the TO element, and so hairpin transcription is uninhibited. Following removal of doxycycline from the system, the process is reversed, and inhibition of hairpin transcription is restored. **(B):** Immunoblot analysis of eight Shef4 TetR subclones for Tetr expression. OCT4 protein was also stained as a marker of the undifferentiated state. The TetR5 clone was chosen as it demonstrated high expression of both TetR and OCT4. Abbreviations: TetR, tet repressor; TO, tet operator.

To generate a hESC line constitutively expressing TetR protein, Shef4 cells were transfected with the pCAG-TetRnls construct (Fig. 1A). Following selection with puromycin, individual clones were expanded and assessed for expression of TetR protein and also for OCT4, indicative of an undifferentiated phenotype (Fig. 1B). Because TetR protein is required for the transcriptional block of hairpin synthesis in this system, it was desirable to select a clone with strong expression of TetR to impair the possibility of leaky expression. Of the eight clones initially selected, clone 5 (Shef4 TetR5) was found to highly express both TetR and OCT4 and was used in subsequent studies. The cell line had similar growth characteristics to the parent line and on differentiation as embryoid body displayed increased expression for genes associated with the three germ layers (supporting information Fig. 1).

### Inducible Knockdown of OCT4 in Shef4 TetR5 Cells

Shef4 TetR5 cells were transfected with the pSUPERIOR vector, into which the OCT4 target hairpin sequence had been cloned (pSUPERIOR-OCT4), and selected with G418. Twenty clones were screened by RT-PCR for effective OCT4 knockdown following treatment with doxycycline. From this screen, three clones provided >80% knockdown (data not shown). The clone that provided the strongest knockdown was used in all subsequent experiments.

**Figure 2:**
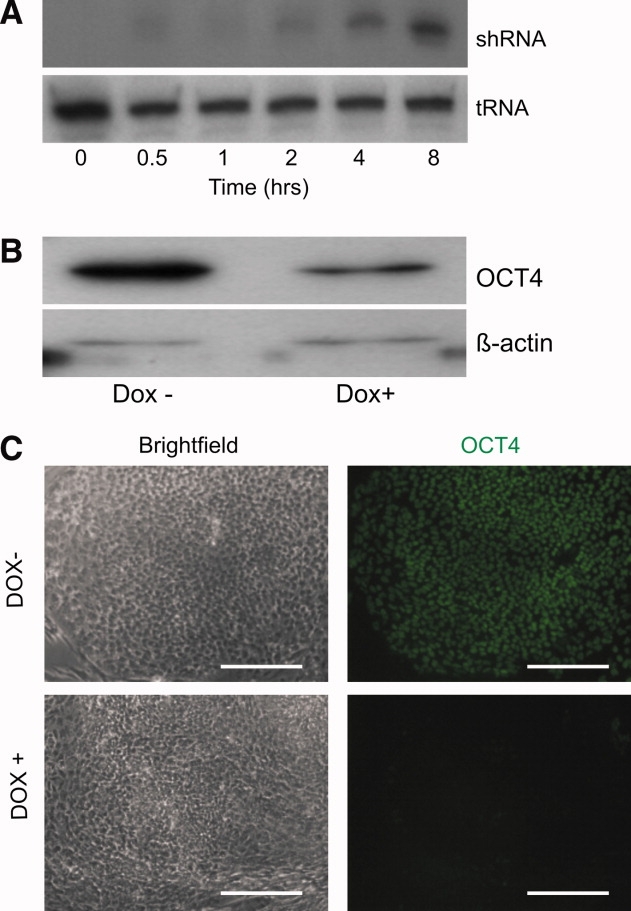
Induction of OCT4-specific hairpin transcription results in OCT4 knockdown. **(A):** Northern blot showing expression of OCT4-specific RNA hairpins following addition of dox to the culture. **(B):** Whole cell lysates of Shef4 TetR5 OCT4-shRNAi cells were stained for OCT4 and β-actin (loading control) following 3 days −/+ dox. **(C):** OCT4-shRNAi interference cells were cultured for 5 days in the absence (top panels) or presence (lower panels) of 5 ng/ml dox. Expression of OCT4 (right panels) is severely reduced by culture with dox. Scale bar: ∼250 μm. Abbreviations: dox, doxycycline; shRNAi, short hairpin RNA; tRNA, transfer RNA.

**Figure 3:**
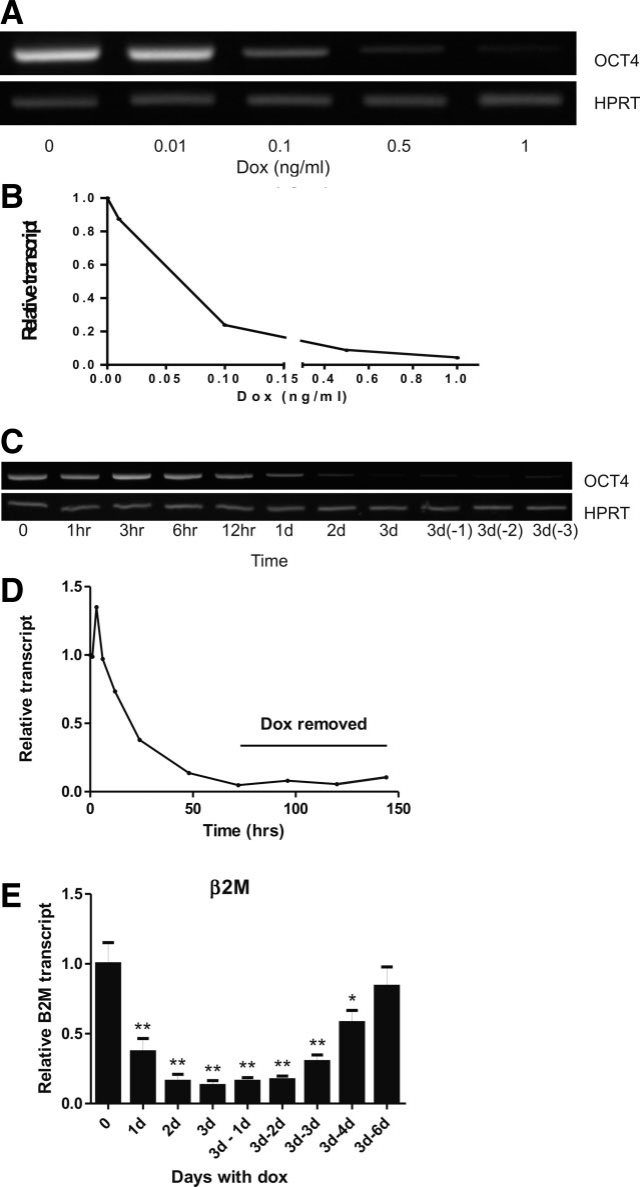
OCT4 target downregulation is dose- and time dependant. **(A):** Reverse transcription-polymerase chain reaction (RT-PCR) analysis of OCT4 and HPRT (loading control) was performed on cells cultured for 3 days with varying concentrations of dox. **(B):** These data are represented graphically and show a steady decrease in OCT4 expression with increasing concentrations of dox. **(C):** Time trial analysis of OCT4 expression by RT-PCR following treatment with dox. After 3-days time point dox was removed form the culture. **(D):** Graphical representation of the RT-PCR data showing maximal knockdown between 2 and 3 days of induction. Following removal of dox, OCT4 expression does not revive due to irreversible differentiation of cell cultures. **(E):** Real time RT-PCR analysis of β2M expression in β2M-shRNAi clone cells, using HPRT as the reference gene control. Cells were cultured for up to 3 days with dox and a further 6 days in the absence of dox, showing recovery of β2M expression. *t* test analysis was performed against dox untreated cells. *, *p* > .05; **, *p* > .005. Abbreviations: β2M, β2-microglobulin; d, day; dox, doxycycline; HPRT, hypoxanthine-guanine phosphoribosyl transferase; hr, hour.

Northern blot analysis of OCT4 hairpin RNA expression confirmed that the system was active and was induced by the addition of doxycycline to the cells (Fig. 2A). At 0 hours, OCT4 hairpin was undetectable, present in low amounts after 0.5 hours, and steadily increased over 8 hours. This production of the hairpin RNA translated into efficient knockdown of OCT4 protein (Fig. 2B, 2C), as indicated by greatly reduced OCT4 levels in whole cell extracts taken from OCT4-shRNAi clone cells induced for 3 days with doxycycline (Fig. 2B) and by immunocytochemistry in which OCT4 protein is undetected following 5 days doxycycline treatment (Fig. 2C). Maximal knockdown of OCT4 at the RNA level was achieved when using doxycycline in excess of 1 ng/ml (data not shown), but the degree of knockdown could be easily manipulated at lower concentrations (Fig. 3A, 3B); the level of induced knockdown was most sensitive between 0.01 and 0.1 ng/ml doxycycline.

Knockdown of the target OCT4 transcripts was also time dependant. A substantial reduction in OCT4 was apparent after 48 hours, while maximal knockdown occurred at 3 days (Fig. 3C, 3D). OCT4 transcript levels still remained completely suppressed 3 days after removal of doxycycline, consistent with the tight linkage of OCT4 expression to the differentiation state of hESCs, the effect of knocking down OCT4 being to induce hESC differentiation. As a target gene control, we carried out an equivalent knockdown using a β2M-shRNAi hESC clone (Fig. 3E); β2M was selected as it is unlikely to influence hESC behavior [27]. The kinetics of β2M transcript downregulation also showed steady downregulation up to 3 days exposure to doxycycline. In this case, it was possible to restore β2M transcript levels following the removal of doxycycline from the culture (Fig. 3E). However, β2M expression only returned to control levels 6 days after the end of doxycycline treatment, which may reflect the half-life of the hairpin, or the time for doxycycline to be cleared from the system, as it is active at very low levels.

### Knockdown of OCT4 by Inducible shRNAi Induces Differentiation of hESCs

**Figure 4:**
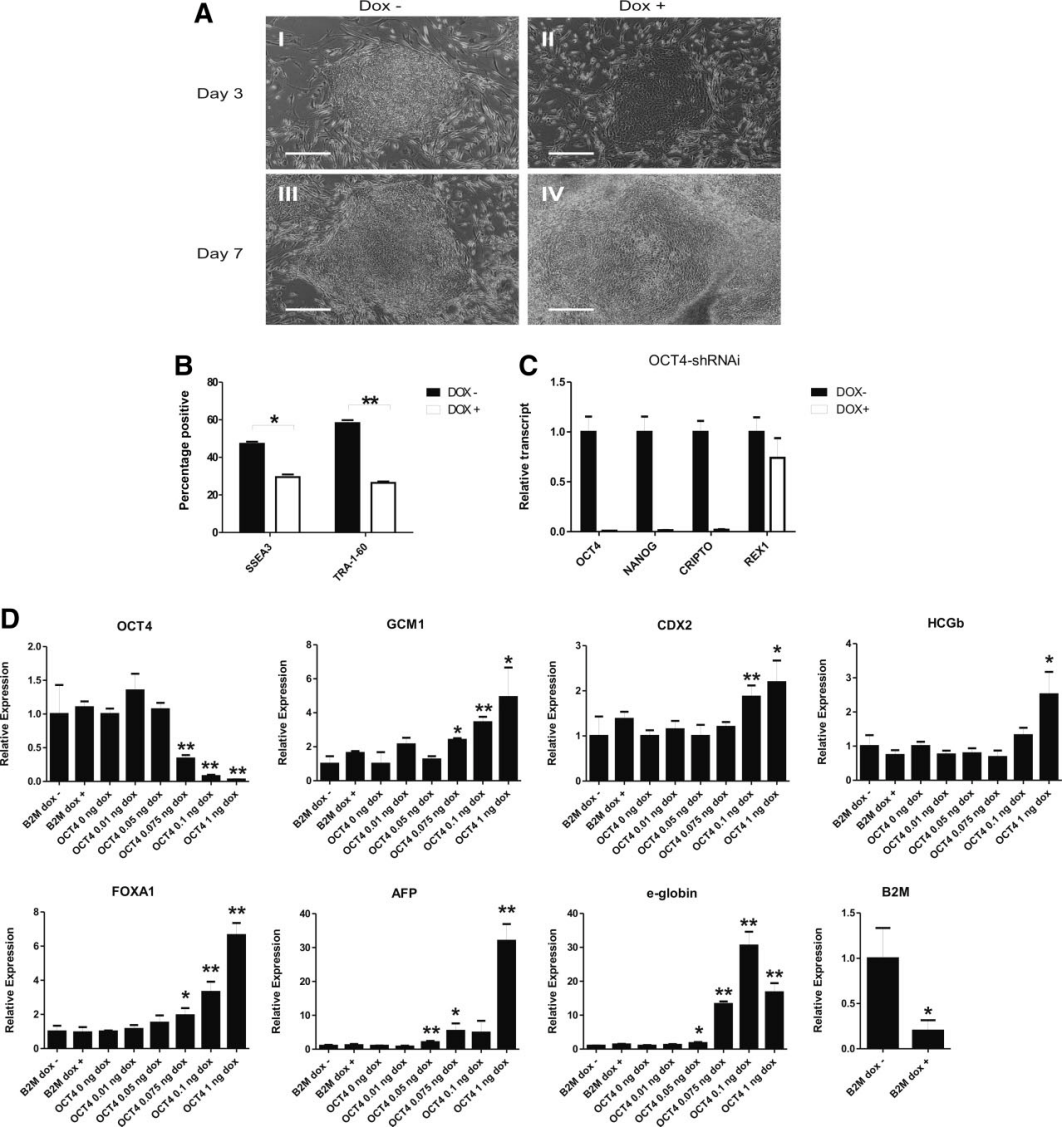
OCT4 downregulation results in cell differentiation and a dose-dependant increase in transcription of lineage specific genes. **(A):** Phase contrast images of Shef4Tetr5 OCT4-shRNAi colonies showing morphological differentiation following doxycyline-induced downregulation of OCT4. Images were taken of colonies 3 days **(I, II)** and 7 days **(III, IV)** postseeding in the absence **(I, III)** or presence **(II, IV)** of doxycyline. White bars: ∼500 μm. **(B):** Flow cytometric analysis of day 7 cultures from noninduced and dox-induced cells shows OCT4 knockdown results in loss of stem cell surface marker expression. The percentage of cells expressing surface markers of the undifferentiated state SSEA3 and TRA-1-60 was significantly reduced (*, *p* > .05; **, *p* > .005) following dox-induced OCT4 downregulation. Results are taken from three separate experiments. **(C):** Real-time reverse transcription-polymerase chain reaction analysis showing reduced transcription of pluripotency associated genes in OCT4 knockdown cells. **(D):** β2M-shRNAi Shef4 cells were cultured for 7 days in absence or presence of dox at 1 ng/ml and OCT-shRNAi Shef4 cells in the presence of doxcycyline from 0 to 1 ng/ml. RNA samples prepared from the cells were used for real time PCR analysis against a panel of genes expressed in differentiated cells types. β2M knockdown was also confirmed in the β2M-shRNAi cells. Data are relative to the samples from 0 ng dox, using hypoxanthine-guanine phosphoribosyl transferase as a reference gene. *t* test analysis was performed against relevant dox untreated cells. *, *p* > .05; **, *p* > .005. Abbreviations: dox, doxycycline; shRNAi, short hairpin RNA interference; SSEA3, stage-specific embryonic antigen-3.

Doxycycline-induced OCT4 knockdown in hESCs resulted in their flattened and more loosely packed appearance (Fig. 4A), which correlated with reduced expression of SSEA3 and TRA-1-60 pluripotent antibody markers (Fig. 4B) and molecular markers NANOG, CRIPTO, and REX1. In contrast, increasing expression was seen for transcripts of trophoblast-associated factors, GCM1, CDX2, and HCGβ when the level of OCT4 was reduced in response to increased (0-1 ng/ml) doxycycline supplementation (Fig. 4D). Likewise, there was increased expression of extraembryonic endoderm and mesoderm-associated genes FOXA1 (formally HNF3α), alpha-fetoprotein, and ɛ-GLOBIN. Doxycycline treatment of β2M-shRNAi cells induced efficient knockdown of β2M; however, this did not result in modification of OCT4 expression or the expression of genes associated with differentiation. These results demonstrate that transcription of markers from both extraembryonic and trophoblast lineages increased by induced OCT4 knockdown, and that the extent of induction can be regulated by the degree knockdown.

## DISCUSSION

Our results show that long-term target gene downregulation can be effectively achieved by inducible shRNAi in hESCs, in a time- and dose-dependent fashion with minimal effect on the phenotype of the undifferentiated hESC cultures in the absence of the inducer. By targeting OCT4, a graded response in the degree of differentiation toward trophoblast and extraembryonic lineage was achieved, which was related to the degree of OCT4 suppression. The data support previous OCT4 knockdown studies, performed on hESCs by transient siRNA and shRNAi [4,16,26,27]; however, the inducible system offers tight control over the level of OCT4 knockdown. By manipulating the concentration of doxycycline, the degree of differentiation could be modulated, as reflected by the expression level of specific factors. We also observed an inverse correlation between the degree of induced OCT4 knockdown and the expression of FOXA1, a transcription factor involved in the differentiation of ESCs toward endoderm [36]. This is consistent with evidence that OCT4 binds directly to the nuclear binding site of the transcription factor FOXD3, so repressing its transcriptional activation of FOXA1 [37]. Thus, as levels of OCT4 were reduced, more FOXD3 was free to drive the expression of FOXA1 and promote endodermal differentiation.

Although siRNA provides a simple system for target gene knockdown, whereby cells can be transfected in situ with synthetic RNAi molecules, the transfection efficiency of these systems in hESCs is low, though improvements have been made using nucleofection [4]. Nevertheless, even with high efficiency transfection, the cell population remains heterogeneous, making detailed analyses difficult. During our generation of inducible shRNAi clones, there was also a high degree of variation in knockdown between stable clones of the same target sequence (data not shown), most likely due to the number of copies of the shRNAi constructs integrated into individual clones, or to positional effects. However, after picking and expanding specific clones, the inducible system provides for reproducible knockdown that is consistent throughout the culture. Moreover, by generating robust clones that are inducible for transgene expression, direct comparisons can be made between cells from the same starting population. This is particularly important when studying hESCs due to the heterogeneity between cultures that arises during even routine passaging.

Constitutive shRNAi has been used previously to study the effect of suppressing OCT4 and SPA-1 (implicated in self-renewal) expression [4,16,17], but the approach was necessarily transient as the hESCs were lost to differentiation before stable clones could be generated. By using the inducible shRNAi system, we have successfully generated a clone that has been cultured in excess of 50 passages, which has retained a normal karyotype, and is primed for efficient OCT4 knockdown by the addition of doxycycline. As the timing of the gene manipulation can be controlled, it is possible to manipulate gene regulation at any specific developmental stage. Furthermore, although OCT4 knockdown could not be reversed because of the differentiation that resulted, we were able to resume β2M expression in the β2M knockdown clones by removal of doxycycline, illustrating the potential to switch gene expression on and off in a development stage-specific manner.

Although other Tet expression based inducible systems have been used both in mouse and human ESCs, there were problems with toxicity [8,38,39]. This most likely relates to the tTA and rtTA transgenes, which were formed by the fusion of the TetR to the transactivating domain of VP16 from Herpes simplex virus. Because our system utilized the wild-type TetR alone without the added transactivating domain, there was no obvious toxicity when generating TetR transgenic clones. Transgene silencing is also an obstacle when generating long-term stable clones [22,40], most likely due progressive silencing of the transgene promoter through prolonged culture. We therefore utilized the pCAG promoter to drive expression of the TetR transgene, a promoter that we have previously shown to retain the expression of eGFP over 120 passages [22]. We found that cells maintained expression of TetR in excess of 100 passages following the withdrawal of puromycin selection.

## SUMMARY

The tet-inducible shRNAi system that we have described and illustrated using OCT4 and β2M knockdown provides a tool by which the levels of specific genes can be regulated robustly in hESCs, directly controlled by the level of induced knockdown. Furthermore, the regulation is reversible. It is a tool that could facilitate the detailed dissection of regulatory systems in the inevitably complex signaling environment that exists in hESC cultures and thereby assist elucidation of the mechanisms that control hESC self-renewal and differentiation.

## DISCLOSURE OF POTENTIAL CONFLICTS OF INTEREST

The authors indicate no potential conflicts of interest.
