# Supplementary figures and images for: Specific Knockdown of OCT4 in Human Embryonic Stem Cells by Inducible Short Hairpin RNA Interference
Source: Stem Cells. 2009 Apr;27(4):776–82. doi: 10.1002/stem.5 (PMC2847189; doi:10.1002/stem.5)

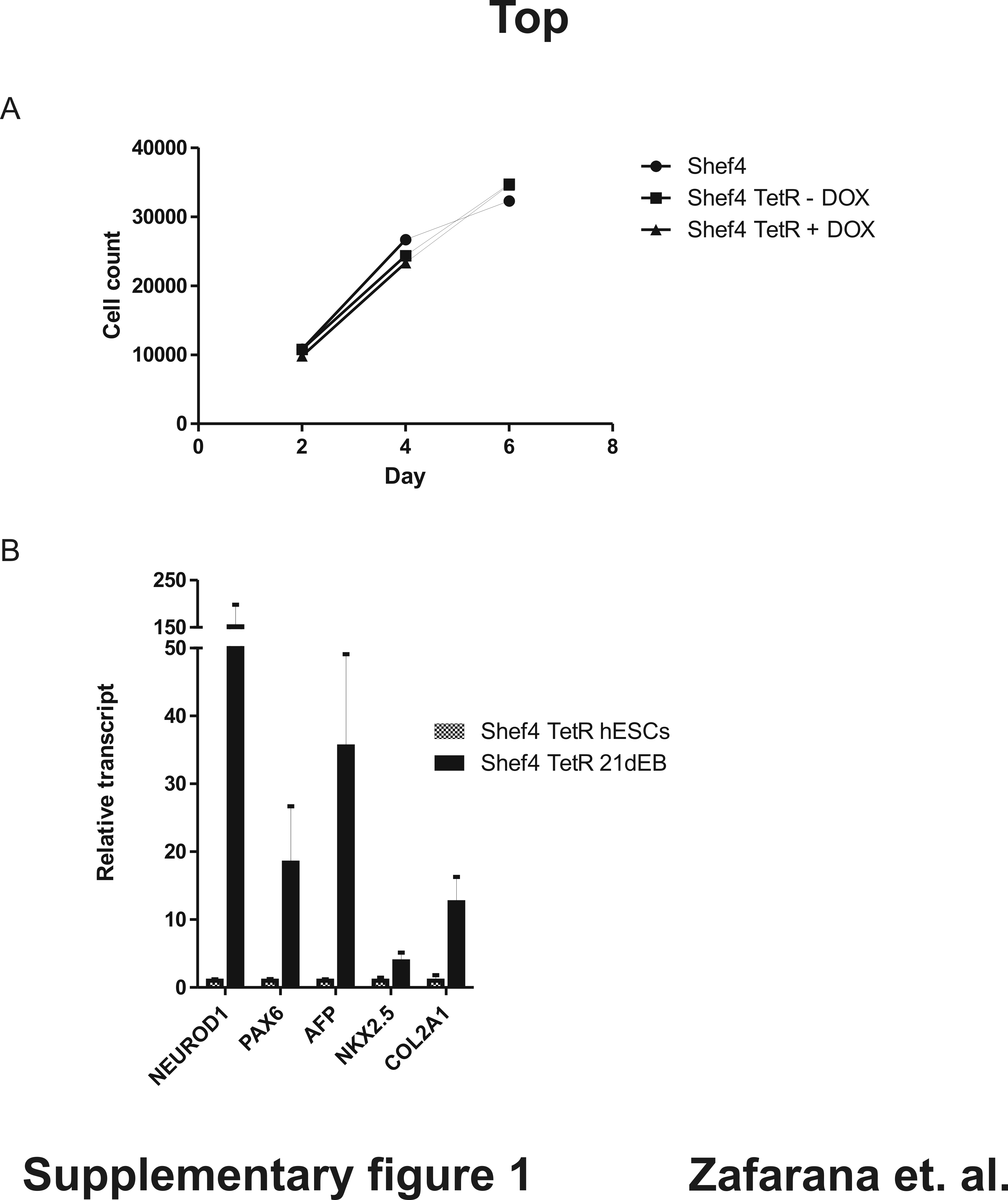

Supplement: Supplementary file 1 [file stem0027-0776-SD1.tif]
